# Effect of adhesive primer on dentin mineral recovery by an alkasite compared to a glass hybrid restorative material: an in vitro study

**DOI:** 10.1186/s12903-026-09973-1

**Published:** 2026-11-05

**Authors:** Yasmin Labib Nasr, Dina Mohamed Ali, Ahmed Fawzy Aboelezz

**Affiliations:** 1 Conservative Dentistry Department, Faculty of Dentistry, Suez Canal University, Ismailia, Egypt; 2 Conservative Dentistry Department, Faculty of Dentistry, October University for Modern Sciences and Arts (MSA), Giza, Egypt

**Keywords:** Bioactive restorative materials, Ion release, pH cycling, Surface microhardness, EDX analysis, Mineral recovery

## Abstract

**Background:**

Bioactive restorative materials can promote dentin mineral recovery, but their efficacy may be influenced by adhesive interfaces. This study aimed to evaluate the effect of an adhesive primer on the mineral recovery potential of an alkasite material (Cention Forte) compared to a glass hybrid material (Equia Forte HT) on artificially demineralized dentin.

**Methods:**

Forty-two human molars with standardized Class V cavities were chemically demineralized and randomly divided into three restorative groups (*n* = 14): Cention Forte without primer (CF-P), Cention Forte with primer (CF + P), and Equia Forte HT (EF). After restoration, all samples underwent 14 days of pH cycling. Mineral recovery was evaluated using Vickers microhardness and energy-dispersive X-ray spectroscopy (EDX) at baseline (T_0_), after demineralization (T_1_), and after pH cycling (T_2_).

**Results:**

All groups demonstrated significant longitudinal increases in absolute microhardness (*p* < 0.0001), following statistically comparable trajectories across materials (interaction, *p* = 0.748). Net microhardness recovery percentages were 74.21% ± 2.48% (CF-P), 72.50% ± 2.10% (EF), and 61.36% ± 3.22% (CF + P). In contrast, the elemental surface profile revealed material-specific differences in deposition with significant interactions (*p* < 0.01). Net calcium recovery was significantly lower in CF + P (68.91% ± 3.66%) than in CF-P (76.10% ± 5.58%) and EF (75.44% ± 2.85%; *p* < 0.05). Phosphorus recovery followed a matching pattern of primer-mediated attenuation (*p* < 0.05). The Ca/P ratio increased significantly in CF-P (*p* = 0.0005) and EF (*p* < 0.0001) but stalled in CF + P (*p* = 0.114).

**Conclusion:**

All restorative protocols achieved statistically comparable mechanical surface stabilization, though their underlying structural pathways diverged. The application of an adhesive primer significantly reduced the surface mineral recovery capacity of the alkasite material. Unprimed Cention Forte (CF-P) demonstrated comparable elemental deposition to the glass hybrid (EF), whereas primer application (CF + P) restricted localized ion-mediated precipitation.

## Introduction

Dental caries remains the most prevalent chronic oral disease globally, with secondary caries at the restoration margin persisting as a leading cause of restoration failure despite continuous advancements in adhesive technology [1, 2]. Contemporary restorative dentistry emphasizes minimally invasive approaches, advocating selective removal of infected dentin while preserving the demineralized, structurally compromised, but potentially remineralizable caries-affected dentin substrate [3]. Facilitating mineral recovery within this residual dentin is important for improving the restoration-tooth interface seal, restoring mechanical integrity, and potentially extending restoration longevity [4].

To support mineral recovery processes, modern restorative biomaterials have evolved from bio-inert formulations toward bioactive systems capable of releasing therapeutic ions such as calcium (Ca²⁺), phosphate (PO₄³⁻), and fluoride (F⁻) into the surrounding microenvironment [5]. The release of these ions may contribute to ionic supersaturation and promote mineral precipitation at the dentin interface. Conventional glass ionomer cements (GICs) have historically served as the principal ion-releasing restorative materials; however, their relatively limited wear resistance and mechanical properties prompted the development of reinforced glass hybrid systems [6]. Equia Forte HT represents an advanced material within this category, incorporating a highly reactive fluoroaluminosilicate glass formulation designed to improve mechanical performance while maintaining sustained ion-release behavior [7, 8].

A different bioactive strategy is represented by alkasite-based restorative materials such as Cention Forte. This material contains alkaline glass fillers capable of releasing Ca²⁺, F⁻, and hydroxyl (OH⁻) ions. The release of OH⁻ ions may be of particular importance during acidic challenges, as localized pH elevation can help neutralize acidic conditions and create an environment favorable for calcium phosphate precipitation [9]. Clinically, the application protocol for this alkasite system involves the use of a proprietary self-etch adhesive primer (Cention Primer) to improve adhesion to dentin [10].

However, the placement of a resin-based adhesive layer may introduce an important physicochemical variable that could influence the bioactivity of ion-releasing restorative materials. Polymerized adhesive interfaces form resin-rich hybrid layers that reduce dentin permeability and may alter ion diffusion dynamics at the restoration interface [11]. Although these adhesive layers are necessary for micromechanical retention, they may also limit the transport of therapeutic ions from the restorative material to the underlying demineralized dentin substrate. Consequently, the extent to which a proprietary adhesive primer affects the mineral recovery behavior of alkasite materials remains an important unresolved question.

Currently, direct comparative data evaluating the mineral recovery performance of Cention Forte with and without its proprietary primer, particularly using the newer capsulated formulation rather than the earlier powder–liquid Cention N system, remain limited. Previous investigations assessing primer effects on alkasite materials have largely focused on isolated fluoride release, evaluated earlier formulations, or lacked assessment of functional mineral incorporation into dentin under dynamic pH-cycling conditions. Furthermore, limited evidence is available comparing these primed and unprimed alkasite protocols with reinforced glass hybrid materials such as Equia Forte HT using combined mechanical and elemental analyses.

Therefore, the present in vitro study aimed to evaluate the influence of an adhesive primer on the mineral recovery behavior of an alkasite restorative material by comparing Cention Forte with and without primer application to a glass hybrid restorative material (Equia Forte HT) under dynamic pH-cycling conditions. Mineral recovery was assessed using surface microhardness testing and energy-dispersive X-ray spectroscopy (EDX) elemental analysis. The null hypothesis was that no significant differences in mechanical or elemental mineral recovery would exist among the three tested restorative protocols (CF − P: Cention Forte without primer; CF + P: Cention Forte with primer; EF: Equia Forte HT).

## Methods

This in vitro study compared three restorative groups: Equia Forte HT (EF; GC Corporation) and Cention Forte (Ivoclar Vivadent) evaluated both without its dedicated primer (CF-P) and with its dedicated primer (CF + P). Details concerning the materials, including their descriptions, compositions, manufacturers, and lot numbers, are provided in Table 1.

**Table 1:** The materials’ trade name, description, composition and manufacturer

Material (Trade Name)	Description	Composition	Manufacturer	Lot no.
Cention^®^ Forte	Bioactive capsulated restorative material	Ca-fluorosilicate glass, Ba–Al silicate glass, Ca–Ba–Al fluorosilicate glass, copolymer, UDMA, aromatic aliphatic UDMA, DCP, PEG-400-DMA, ytterbium trifluoride	Ivoclar Vivadent AG, Schaan, Liechtenstein	ZL08SR
Cention^®^ Primer	Self-etching, self-curing primer for tooth structure	HEMA, MDP, Bis-GMA, D3MA, ethanol, methacrylate-modified polyacrylic acid, silicon dioxide, potassium hydroxide, camphorquinone	Ivoclar Vivadent AG, Schaan, Liechtenstein	Z065VK
Equia Forte^®^ HT	Bulk-fill, fluoride-releasing glass hybrid restorative system	Powder: Fluoroaluminosilicate glass, polyacrylic acid, iron oxide Liquid: Polybasic carboxylic acid, water	GC Corporation, Tokyo, Japan	2,201,052

### Sample size calculation

This in vitro study was approved by the Ethics Committee of the Faculty of Dentistry, Suez Canal University (Approval No. 623/2023). The requirement for informed consent was waived because the study used anonymized, extracted human molars. Sample size was calculated a priori using G*Power software (version 3.1.9.6) for a one-way ANOVA design [12, 13]. An effect size of f = 0.50 was selected based on prior in vitro studies evaluating bioactive restorative materials, where intergroup contrasts consistently exceeded conventional thresholds for large effects [14, 15].

A minimum total sample of 42 specimens (*n* = 14 per group: CF-P, CF + P, EF) was determined to be sufficient to achieve 80% statistical power (1-β = 0.80) at α = 0.05.

While longitudinal absolute measurements were tracked across T₀, T₁, and T₂, the cross-sectional hypotheses utilized derived recovery percentages—single independent endpoints per specimen that integrate the full measurement arc—rendering the design consistent with the one-way ANOVA model. Both microhardness and EDX recovery percentages were analyzed within the same statistical framework to allow parallel evaluation of mechanical and elemental outcome patterns.

The experimental timeline comprised three distinct assessment intervals (T₀, T₁, and T₂) for both microhardness and elemental analysis. Baseline measurements (T₀) were obtained directly following cavity preparation. Specimens were evaluated a second time (T₁) following chemical demineralization induction, and a final evaluation (T₂) was conducted after completing the respective restorative procedures and dynamic pH-cycling protocols.

**Fig. 1:**
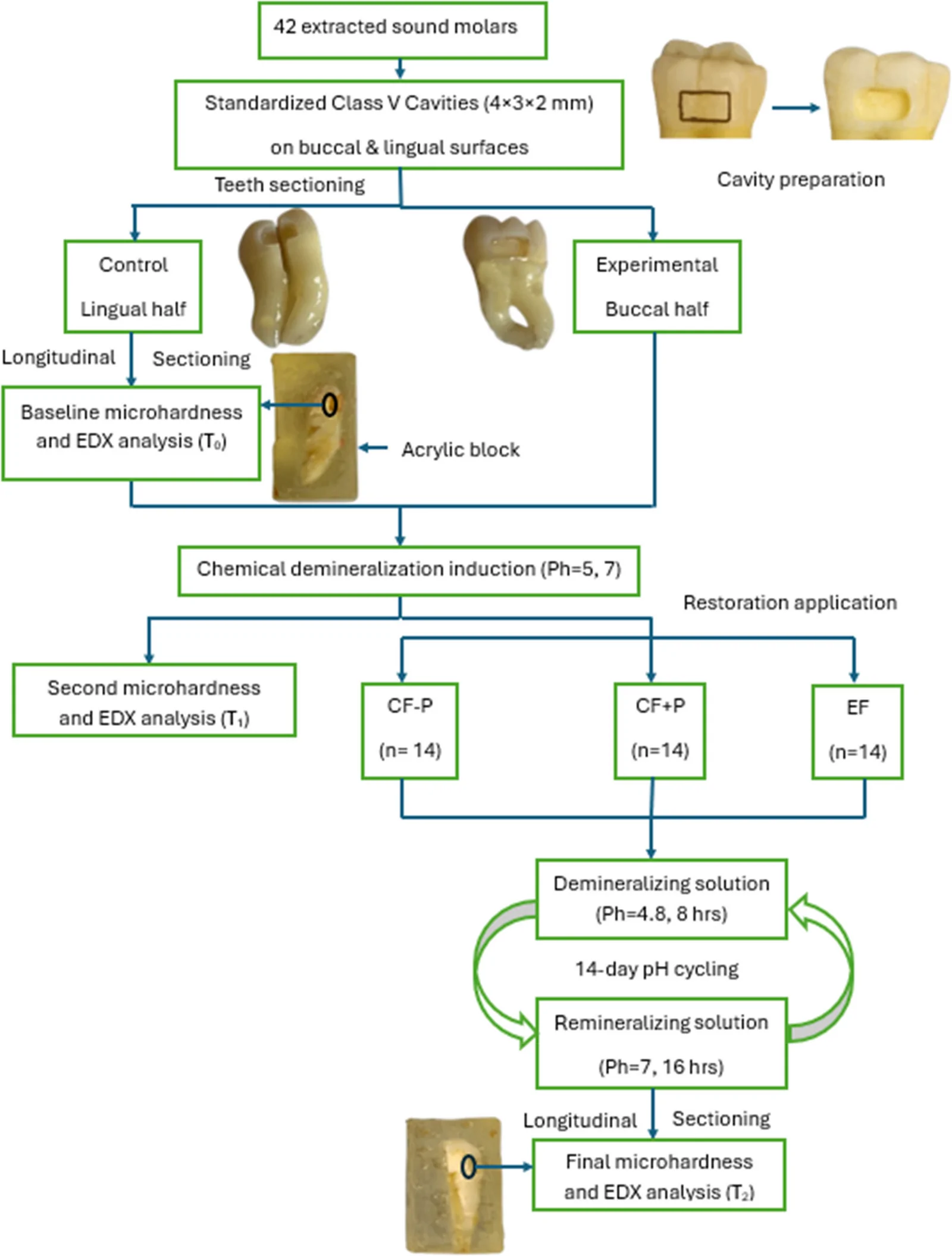
Specimen preparation flowchart of the study design

The specimen preparation procedure and the study workflow are depicted in Fig. 1.

### Sample preparation

Standardized Class V cavities were prepared on both the buccal and lingual surfaces of each tooth using a size #57 straight carbide fissure bur (Meisinger GmbH, Germany) mounted in a high-speed handpiece under copious water cooling. To ensure cavity uniformity and consistent positioning, a metal matrix band with a 4 mm × 3 mm window was secured to each surface using a Tofflemire retainer (ASA Dental GmbH, Germany). The outline of the cavity was marked with a pencil, with the lower edge of the matrix band aligned with the cementoenamel junction (CEJ) and the window’s lower margin positioned 1 mm above the CEJ [16].

The bur was replaced after every fifth preparation to maintain cutting efficiency and standardization. Each cavity was prepared with the following dimensions: 4 mm width (mesiodistally) × 3 mm height (occlusogingivally) × 2 mm depth, and positioned parallel to the CEJ, extending 1 mm above it [17]. A graduated periodontal probe (Williams 568/1, Medesy, Italy) was used to verify the dimensions of each cavity, ensuring consistency across all specimens [18].

To enhance reproducibility and minimize experimental bias, standardized procedures were maintained throughout specimen preparation and testing. The specimens were randomly assigned to the experimental groups (*n* = 14) using a computer-generated randomization sequence (Random.org). Each specimen container was assigned an alphanumeric code prior to analysis to facilitate standardized handling and data recording. All restorative procedures, pH-cycling protocols, and analytical assessments were performed by a single trained operator to eliminate inter-operator procedural variability. Although operator blinding was not feasible due to distinct material application protocols, surface microhardness and EDX analyses were conducted using automated instrument parameters and predefined digital measurement boundaries to eliminate subjective operator bias.

### Baseline microhardness evaluation

Each tooth was longitudinally sectioned in the occluso-gingival direction using a low-speed diamond saw under continuous water cooling, yielding two halves: buccal and lingual. To ensure consistency in baseline mineral composition between both halves of the same tooth, elemental analysis was performed on the pulpal floor of the buccal and lingual sections in a representative sample. This analysis confirmed the absence of significant differences in mineral content between the two halves. Each half was individually labeled to maintain accurate identification and traceability throughout subsequent treatment and testing procedures.

The lingual cavity surface of each tooth was further sectioned longitudinally through its center using a slow-speed diamond saw under water cooling, producing two quadrants. In this study design, the lingual half of each tooth functioned as the control, while the buccal half served as the experimental (test) group [19].

One quadrant from each control sample was randomly selected and embedded in self-curing acrylic resin within a plastic ring. The embedded surfaces were then progressively refined and polished using a series of flexible abrasive discs (Sof-Lex™, 3 M ESPE) to obtain a smooth and standardized surface suitable for microhardness testing.

Baseline cross-sectional microhardness with a Vickers diamond indenter was carried out for all experimental groups. The microhardness assessments were carried out using a Vickers’ Microhardness Tester, utilizing a Vickers’ diamond indenter and a 20X objective lens. Readings were recorded at three distinct locations on each specimen: on the axial dentin, approximately 0.5 mm from the preparation margin, and spaced 1 mm apart from each other. Each reading was performed using a 100 g load for a duration of 15 s, with the load-oriented perpendicular to the dentin surface. The diagonal lengths of the resulting indentations were measured using an incorporated scaled micrometer, and these measurements were converted into Vickers numbers. The obtained values were averaged to generate a singular hardness value for each individual specimen. Micro-hardness was calculated using the subsequent equation: HVN = 1.854 P/d2, where HVN is Vickers hardness in kgf/mm², P is the applied load in kgf, and d is the mean diagonal length in mm [18].

### Elemental analysis

The remaining quadrant of each lingual cavity was used for elemental analysis using Energy-Dispersive X-ray Spectroscopy (EDX). The system was operated through Inca Energy software, version 18. Prior to imaging, the dentin surfaces were treated with 17% ethylenediaminetetraacetic acid (EDTA) for 3 min, followed by immersion in 2% sodium hypochlorite (NaClO) for an additional 3 min, to eliminate the smear layer [20].

The specimens were mounted on aluminum stubs using carbon double-sided adhesive tape. Imaging and elemental analysis were conducted under low vacuum conditions (100 Pa) using an ESEM/EDX. Operating parameters included an accelerating voltage of 20 kV, a working distance of 8.5 mm, a detection threshold of 0.5 wt%, a resolution of 133 eV, and an amplification time of 100 microseconds. Data acquisition was performed over 600 s for elemental mapping and 60 s for spectral collection. Analysis was carried out over areas of approximately 50 × 50 μm, allowing for the quantification of the elemental composition specifically calcium (Ca), phosphorus (P), oxygen (O), and carbon (C) expressed in both weight and atomic percentages. The Ca/P ratio was calculated from the weight% EDX data to assess the mineral phase characteristics of the dentin surface [21].

### Chemical demineralization induction

The prepared buccal cavities and control lingual quadrants were first rinsed with deionized water and gently air-dried. To isolate the experimental area, two coats of acid-resistant nail varnish were applied to all surfaces except for a window that included the cavity and a 2 mm border of sound surrounding tooth structure. The first coat was allowed to air-dry at room temperature for 3–4 h before the second coat was applied. To induce artificial demineralization, the exposed window areas of all three groups were immersed in a chemical caries-inducing solution for 7 days, using 50 mL of solution per sample. The demineralizing medium consisted of a 50 mM acetate buffer containing 2.2 mM CaCl₂ and 2.2 mM KH₂PO₄, adjusted to pH 5. The acetate-buffer system at pH 5.0 has been widely employed for lesion induction because it generates reproducible subsurface mineral loss while preserving the dentin matrix, thereby providing a standardized baseline for subsequent evaluation of restorative materials [22].

The solution was freshly prepared each day and replaced every 24 h to maintain consistent chemical conditions. After 7 days of demineralization, specimens were removed and rinsed thoroughly with deionized water for 3 min, followed by ultrasonic cleaning in deionized water for 5 min to stop the demineralization process and remove residual solution.

### Second evaluation following demineralization

Following the induction of artificial caries-like lesions, the control specimens which had previously undergone baseline assessment for Vickers cross-sectional microhardness and elemental composition were re-evaluated using the same testing protocols. This second round of measurements was conducted to obtain the corresponding post-demineralization values, allowing for comparison with baseline data and assessment of mineral loss resulting from the demineralization process.

### Restorative material application

For each of the three experimental groups—Cention Forte without primer (CF-P), Cention Forte with primer (CF + P), and Equia Forte HT (EF)—the prepared cavities were thoroughly rinsed with water to remove debris, then dried using sterile cotton and compressed air. Restorative procedures were performed on the buccal surfaces of all teeth (*n* = 14 per group).

The materials were applied in accordance with the manufacturers’ instructions, as follows:

#### CF-P

The capsule was activated and inserted into the mixer for 15s. After mixing, the material was placed in the applicator and dispensed in the cavity. Light curing was performed for 20s.

#### CF+P

A drop of Cention Primer was dispensed into a dish to protect it from light. The corresponding coated Cention Primer single-use applicator was dipped in the liquid and stirred for 5s. The mixing process was complete when the applicator had a uniform yellow color. The cavity surface was scrubbed with the primer for 10s. Air thinning was performed until a shiny, thin, immobile film had formed. The capsule was activated and inserted into the mixer for 15s. After mixing, the material was placed in the applicator and dispensed in the cavity. Light curing was performed for 20s.

#### EF

The capsule was activated and inserted in the mixer for 10 s. After mixing, the material was placed in the applicator and dispensed in the cavity. Light curing was performed for 20s.

All specimens were polymerized using a LED light-curing unit (Elipar™, 3 M ESPE, St. Paul, MN, USA) operating in standard mode with a light intensity of 1200 mW/cm². To ensure standardized curing, the tip of the curing light was held in direct contact with the buccal surface of each specimen during polymerization.

### pH cycling

To simulate the dynamic process of demineralization and remineralization that occurs in the oral environment, a 14-day pH cycling regimen was applied to all restored specimens. In contrast to the initial static lesion-induction phase, the pH-cycling protocol employed a sodium phosphate-based demineralizing solution at pH 4.8 as part of a validated dynamic demineralization/remineralization model. This model was designed to simulate the cyclic acidic challenges associated with the clinical caries process through alternating demineralization and remineralization phases [23, 24]. The samples were alternately immersed in two solutions: Demineralizing solution: 2.2 mM calcium chloride, 2.2 mM sodium phosphate, and 50 mM acetic acid, adjusted to pH 4.8, for a period of 8 h daily. Remineralizing solution: 1.5 mM calcium chloride, 0.9 mM sodium phosphate, and 0.15 mM potassium chloride, adjusted to pH 7.0, for the remaining 16 h of the day [23]. All specimens were maintained in an incubator at 37 °C under constant circulation throughout the cycling period. To ensure the chemical stability and efficacy of the solutions, freshly prepared demineralization and remineralization media were replaced every 24 h [24].

### Final evaluation following pH cycling

Following pH cycling, the restored buccal cavities from all experimental groups were sectioned in a buccolingual direction using a low-speed diamond saw under water cooling. The resulting specimens were then subjected to the final evaluation of Vickers cross-sectional microhardness and elemental analysis, using the same protocols previously described for baseline and post-demineralization assessments.

### Statistical analysis

Statistical analysis was performed using MedCalc software version 22.0 (MedCalc Software Ltd, Ostend, Belgium). Continuous variables were assessed for normality using the Shapiro–Wilk test. All datasets demonstrated a normal distribution and are presented as mean ± standard deviation. The global significance threshold was set at α = 0.05.

To evaluate the longitudinal trajectories of absolute microhardness and absolute elemental surface data across evaluation intervals (T₁ and T₂), two-way mixed-model ANOVAs were performed with material type as the between-subjects factor and time as the within-subjects repeated factor. Pairwise between-group post hoc comparisons within these mixed models were executed using Bonferroni correction. Longitudinal within-group changes between phases were verified using paired t-tests.

Recovery percentages for microhardness and EDX parameters (calcium, phosphorus, carbon, oxygen, and Ca/P ratio) were calculated individually for each specimen using its corresponding baseline (T₀), post-demineralization (T₁), and post-pH-cycling (T₂) values according to the formula $\:\left[\right({T}_{2}-{T}_{1})/({T}_{0}-{T}_{1}\left)\right]\times\:100$ [25]. Group recovery percentages are presented as the mean ± SD of the individual specimen recovery values. Between-group variations for these derived net recovery percentages were evaluated using separate one-way fixed-effects ANOVAs followed by Tukey’s HSD post hoc tests to isolate material-specific differences.

## Results

### Vickers microhardness

Artificial demineralization (T₁) resulted in a reduction in dentin microhardness across all experimental groups. Following 14 days of pH cycling, absolute microhardness values increased from T₁ (post-demineralization) to T₂ (post-pH cycling) for all tested protocols (*p* < 0.0001; Table 2). Net microhardness recovery percentages were 74.21% ± 2.48% for CF − P, 72.50% ± 2.10% for EF, and 61.36% ± 3.22% for CF + P (Table 3).

**Table 2:** Mean ± standard deviation of microhardness (VHN) and elemental composition (weight%) of dentin at baseline, after demineralization (T₁) and after pH cycling (T₂) for each restorative group

	Group	Baseline (T₀)	Post-Demineralization (T₁)	Post-pH Cycling (T₂)	*p*-value (T₁ vs. T₂)*
VHN	CF − *P*	53.62 ± 1.67	47.74 ± 2.36	52.11 ± 1.69	< 0.0001
	CF + P	53.90 ± 1.80	47.69 ± 2.68	51.51 ± 1.95	< 0.0001
	EF	54.11 ± 1.50	47.72 ± 1.70	52.36 ± 1.56	< 0.0001
Ca (wt%)	CF − P	29.41 ± 1.78	15.11 ± 4.12	25.99 ± 1.22	< 0.0001
	CF + P	31.48 ± 1.83	22.90 ± 3.64	28.79 ± 2.11	< 0.0001
	EF	30.79 ± 1.23	18.49 ± 3.57	27.75 ± 1.59	< 0.0001
P (wt%)	CF − P	12.86 ± 0.40	7.93 ± 1.72	11.54 ± 0.48	< 0.0001
	CF + P	14.36 ± 0.40	10.84 ± 1.55	13.01 ± 0.66	< 0.0001
	EF	13.88 ± 0.35	11.84 ± 1.64	13.24 ± 0.70	< 0.0001
Ca/P Ratio	CF − P	2.28 ± 0.17	1.89 ± 0.19	2.25 ± 0.12	0.0005
	CF + P	2.19 ± 0.14	2.11 ± 0.26	2.21 ± 0.14	0.1142
	EF	2.22 ± 0.12	1.59 ± 0.40	2.10 ± 0.20	< 0.0001

*CF−P* Cention Forte without primer, *CF+P* Cention Forte with primer, *EF* Equia Forte HT

*Paired t-test

**Table 3:** Mean ± standard deviation recovery percentages of microhardness and elemental composition for dentin restored with Cention Forte without primer (CF − P), Cention Forte with primer (CF + P), and Equia Forte HT (EF) following pH cycling

	CF − *P*	CF + *P*	EF	*p*-value*
VHN	74.21 ± 2.48	61.36 ± 3.22	72.50 ± 2.10	< 0.001
Calcium (Ca)	76.10 ± 5.58^A^	68.91 ± 3.66^B^	75.44 ± 2.85^A^	< 0.001
Phosphorus (P)	73.15 ± 1.52^A^	61.38 ± 1.99^C^	68.61 ± 2.57^B^	< 0.001
Carbon (C)	73.31 ± 5.34^A^	66.78 ± 4.60^B^	74.08 ± 4.98^A^	< 0.001
Oxygen (O)	77.37 ± 7.09^A^	65.85 ± 7.47^B^	74.28 ± 6.29^A^	< 0.001
Ca/P Ratio	79.98 ± 11.42^A^	60.44 ± 23.63^B^	78.74 ± 2.90^A^	0.009

*One-way ANOVA followed by Tukey’s post-hoc test. Values within the same row sharing a common superscript letter (A, B, C) are not significantly different (*p* > 0.05). Different superscript letters indicate statistically significant differences (*p* < 0.05). For all parameters except Phosphorus (P), groups CF − P and EF (both labeled ‘A’) are not significantly different, but both are significantly higher than CF + P (labeled ‘B’). For Phosphorus, all three groups are significantly different from each other (CF − P > EF > CF + P)

Two-way mixed-model ANOVA revealed a significant main effect of time (F = 92.843, *p* < 0.001), reflecting consistent hardening across all groups over the evaluation period. No significant main effect of material type (F = 0.351, *p* = 0.705) or material × time interaction (F = 0.292, *p* = 0.748) was observed (Table 4).

**Table 4:** Two-way ANOVA results (F-values and p-values) for the effects of restorative material, time (mineralization state), and their interaction on dentin microhardness and elemental composition

Dependent Variable	Source	F-value	*p*-value
Vickers Hardness	Material	0.351	0.705
	Time	92.843	**< 0.001**
	Material × Time	0.292	0.748
Calcium (Ca)	Material	22.890	**< 0.001**
	Time	184.360	**< 0.001**
	Material × Time	5.270	**0.007**
Phosphorus (P)	Material	39.770	**< 0.001**
	Time	78.381	**< 0.001**
	Material × Time	5.737	**0.005**
Ca/P Ratio	Material	11.894	**< 0.001**
	Time	34.833	**< 0.001**
	Material × Time	4.863	**0.010**

*Time refers to the mineralization state (demineralization T₁ vs. remineralization T₂). Bold *p*-values indicate statistical significance (*p* < 0.05)*

### EDX analysis

In contrast to the uniform mechanical recovery profiles, two-way mixed-model ANOVAs revealed significant material × time interactions for calcium (F = 5.270, *p* = 0.007) and phosphorus (F = 5.737, *p* = 0.005) weight percentages (Table 4).

At T₁, the CF + P group presented with the highest residual calcium content (22.90 ± 3.64 wt%; Table 2). Despite this, net calcium recovery was significantly higher in CF − P (76.10% ± 5.58%) and EF (75.44% ± 2.85%) than in CF + P (68.91% ± 3.66%; *p* < 0.001; Table 3). CF − P and EF did not differ significantly from each other.

Net phosphorus recovery was significantly different across all three groups, with CF − P achieving the highest recovery (73.15% ± 1.52%), followed by EF (68.61% ± 2.57%), and CF + P showing the lowest (61.38% ± 1.99%; *p* < 0.001 for all pairwise comparisons; Table 3).

### Ca/P ratio and organic matrix

The Ca/P ratio showed a significant material × time interaction (F = 4.863, *p* = 0.010; Table 4). Following pH cycling, absolute Ca/P ratios increased significantly in CF − P (*p* = 0.0005) and EF (*p* < 0.0001) but showed no significant change in CF + P (*p* = 0.114; Table 2). Net Ca/P ratio recovery was significantly higher in CF − P (79.98% ± 11.42%) and EF (78.74% ± 2.90%) than in CF + P (60.44% ± 23.63%; *p* = 0.009; Table 3). CF − P and EF did not differ significantly from each other.

Carbon and oxygen weight percentages decreased significantly from T₁ to T₂ across all groups (*p* < 0.0001; Table 2). Net recovery percentages for carbon and oxygen reduction were significantly higher in CF − P and EF than in CF + P (*p* < 0.001; Table 3), while CF − P and EF did not differ significantly from each other for carbon. For oxygen, no significant difference was observed between CF − P and EF.

**Fig. 2:**
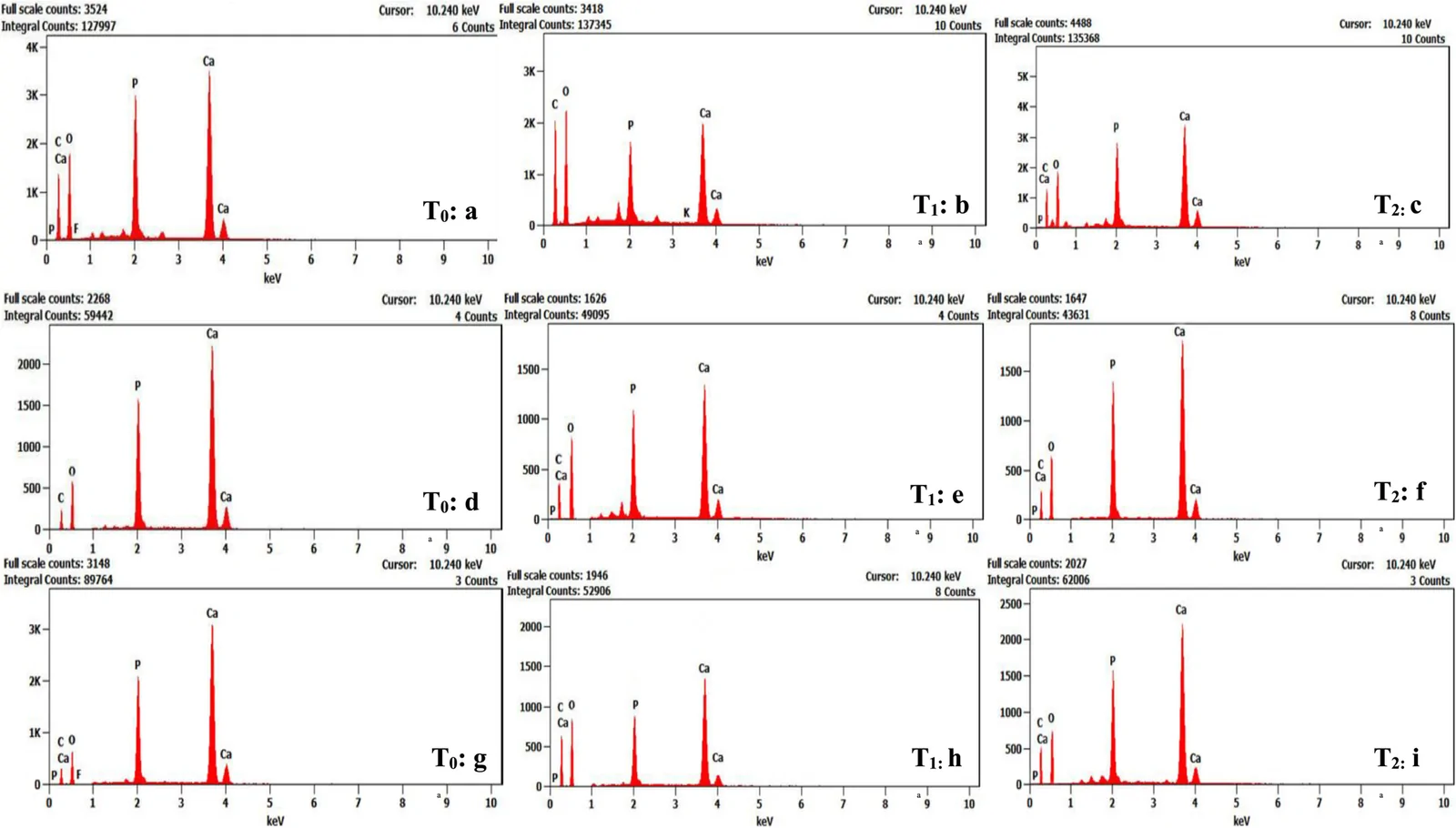
Representative energy-dispersive X-ray (EDX) spectra of dentin adjacent to Cention Forte without primer (CF − P) at baseline (T_0_: a), post-demineralization (T₁: b), and post-pH cycling (T₂: c); Cention Forte with primer (CF + P) at baseline (T_0_: d), post-demineralization (T₁: e), and post-pH cycling (T₂: f); and Equia Forte HT (EF) at baseline (T_0_: g), post-demineralization (T₁: h), and post-pH cycling (T₂: i). Representative spectra demonstrate the reduction in calcium and phosphorus peaks following demineralization and their partial recovery after pH cycling

Representative EDX spectra of demineralized dentin restored with (a) CF-P, (b) CF + P, and (c) EF at baseline (T₀), post-demineralization (T₁), and post-pH cycling (T₂), illustrating relative changes in calcium and phosphorus peak intensities (Fig. 2).

## Discussion

The high global prevalence of dental caries underscores a clinical requirement for restorative materials that provide therapeutic ion exchange in addition to structural replacement [26]. While conventional resin composites demonstrate adequate mechanical and esthetic properties, their bio-inert nature limits their efficacy in high-caries-risk environments. This study evaluated the dentin mineral recovery potential of two distinct bioactive systems: an alkasite material (Cention Forte) and a high-viscosity glass hybrid (Equia Forte HT), focusing on their capacity to support dentin mineral recovery.

Cention Forte was selected on the basis of its alkaline glass filler technology, which is engineered to release Ca²⁺, F⁻, and OH⁻ ions in response to acidic challenges [27]. In the context of a dentin substrate, the release of OH⁻ ions is of particular interest, as the resulting localized pH elevation may stabilize the demineralized collagen matrix and create an environment conducive to calcium phosphate supersaturation [22, 28]. While earlier investigations predominantly evaluated the powder-liquid formulation (Cention N) and demonstrated promising outcomes [29, 30], the capsulated Cention Forte used here provides standardized mixing ratios and improved reproducibility, enabling a more controlled assessment of functional recovery under pH-cycling conditions.

The inclusion of the proprietary primer represents a clinically relevant modification; however, resin-based adhesive systems are known to alter the physicochemical environment at the dentin interface, with acidic functional monomers forming resin-rich interfacial layers that may restrict ion diffusion [11, 30, 31]. Equia Forte HT served as a validated bioactive comparator, with an ion-release profile characterized by an initial burst of F⁻ followed by sustained diffusion of both F⁻ and Ca²⁺ ions, attributed to its fluoroaluminosilicate glass composition [32, 33].

Dentin was selected as the substrate to reflect the clinical complexity of partially demineralized caries-affected tissue. Unlike enamel, which is predominantly inorganic, dentin repair requires processes that address both the organic collagen scaffold and the inorganic mineral phase [22, 28]. To capture this complexity, Vickers microhardness and EDX elemental analysis were combined: microhardness quantifies the functional mechanical recovery of tissue resistance, while EDX provides direct compositional data at the dentin surface.

Crucially, while these combined surface metrics provide a comprehensive evaluation of interfacial dynamics, they collectively reflect physicochemical mineral recovery at the immediate dentin surface rather than biological remineralization in the strict sense. True biological remineralization requires guided hydroxyapatite nucleation deep within the demineralized collagen scaffold mediated by non-collagenous proteins [22, 28]. Kunert et al. similarly emphasized that true dentin remineralization involves intrafibrillar apatite formation within the collagen matrix, a process distinct from surface mineral precipitation [34]. Therefore, these outcomes should be interpreted as validated proxies for mineral deposition potential rather than direct confirmation of intrafibrillar apatite formation or collagen-guided crystal growth.

In particular, while the tracked Ca/P ratio indicates stoichiometric shifts toward baseline values, EDX elemental data alone cannot confirm true crystalline phase formation. Definitive structural confirmation of hydroxyapatite development would require analytical techniques such as X-ray diffraction, Raman spectroscopy, or transmission electron microscopy—characterizations that lie outside the scope of this investigation. Given the clinical importance of bioactive materials in caries management, the findings of this study indicate that while absolute physical hardening occurs uniformly across protocols, the true elemental deposition profile is highly dependent on both material composition and interfacial primer barriers. This nuanced behavior leads to the rejection of the null hypothesis.

The observation that absolute microhardness recovery was statistically comparable across all three groups, while EDX elemental outcomes diverged significantly, warrants explicit reconciliation. One plausible interpretation is that mechanical hardness recovery in the primed group (CF + P) may have been partially influenced by infiltration of the polymerized adhesive primer into the demineralized dentin matrix, forming a resin-reinforced hybrid layer that partially restored tissue resistance through a hybridization mechanism rather than through mineral ion deposition [31, 35].

Under this interpretation, the two recovery mechanisms—resin-mediated mechanical reinforcement and ion-mediated elemental mineral incorporation—are not equivalent proxies but functionally distinct processes [31]. This distinction is clinically significant: mechanical hardness recovery through hybridization does not confer the same long-term biological protective effect as genuine mineral ion incorporation, which supports continued ion exchange and potential acid-buffering capacity [31]. This hypothesis is consistent with the EDX findings showing attenuated calcium and phosphorus recovery in CF + P, and explains why mechanical equivalence does not preclude elemental divergence [36, 37].

When interpreting the mechanical outcomes, it is essential to contextualize the microhardness findings within the underlying statistical model to avoid overstating material-specific efficacy. The two-way mixed-model ANOVA revealed a significant main effect for time, confirming that all three restorative protocols successfully supported surface hardening of the demineralized dentin matrix following the 14-day dynamic pH-cycling regimen. However, the absence of a significant material main effect or material-time interaction establishes that the absolute magnitude and longitudinal trajectory of physical hardness recovery were statistically comparable among the groups.

Clinically, this uniform mechanical recovery underscores the shared functional reliability of these bioactive formulations; whether utilizing an alkasite-based material or a high-viscosity glass hybrid, all three protocols successfully drive a marked recovery of surface mechanical resistance within the complex dentin matrix.

The observed uniform mechanical recovery aligns well with the broader consensus in the literature, where both alkasite and glass hybrid mechanisms are recognized as effective vehicles for mechanical substrate rehabilitation. While historical studies reported localized variations on enamel substrates [14, 38], the present findings demonstrate that when applied to a dentin substrate under dynamic pH-cycling conditions, the multi-ion release and buffering capacity of these materials converge on parallel absolute functional endpoints. This distinction between enamel and dentin remineralization mechanisms is clinically important, as enamel recovery is largely driven by fluoride-mediated surface redeposition, whereas dentin repair requires ion penetration into a collagenous matrix [39].

Furthermore, reported discrepancies in literature rankings regarding the comparative performance of alkasite and glass hybrid materials frequently stem from variations in evaluation depths, substrate parameters, and assessment durations rather than fundamental differences in absolute mechanical potential [15, 40, 41]. For instance, the sustained fluoride-release profile of glass hybrids may optimize mineral recovery over a broader subsurface region at greater depths [41], whereas the immediate local alkaline buffering of unprimed alkasites exerts a pronounced early effect directly at the restoration interface [42]. Because the 14-day evaluation used here represents an intermediate stage of mineral maturation rather than a definitive kinetic endpoint [43, 44], the statistically parallel absolute microhardness trajectories confirm that both systems are equally capable of achieving functional mechanical repair during this evaluation window.

Consequently, while surface microhardness serves as a reliable parallel functional validator of structural recovery, the primary differentiating evidence for material-dependent elemental recovery is carried entirely by the elemental and compositional data. In contrast to the uniform physical recovery profiles, the EDX analysis revealed distinct, material-specific variations in elemental precipitation following pH cycling. The unprimed CF-P group demonstrated the highest absolute calcium recovery, followed closely by EF, while the primed CF + P cohort showed the lowest. This pattern is consistent with prior characterizations of the high calcium ion-release capacity and acid-buffering kinetics of alkasite materials [30, 38, 45]. The fluoride release and recharge capacity of alkasite materials have also been documented, with Cention N demonstrating superior post-recharge fluoride release compared to conventional GICs [46].

While data in the literature have presented higher cumulative calcium release into static solutions from Equia Forte HT compared to an alkasite [32], that apparent discrepancy highlights the difference between theoretical bioactive potential in static models and functional elemental incorporation under dynamic pH-cycling conditions. In a dynamic matrix, the rapid, localized calcium availability and alkaline buffering capacity of the unprimed alkasite may be more effective at driving surface mineral incorporation within the short-term evaluation window.

Phosphorus recovery followed a consistent pattern, with EF performing comparably to CF-P. The robust phosphate-related behavior of Equia Forte HT is consistent with its fluoroaluminosilicate glass composition, which has previously been associated with sustained phosphate ion activity [30]. While prior work reported higher cumulative phosphate release from Cention N compared with a glass ionomer over an extended static period [27], the parity observed between CF-P and EF in the present study reflects phosphorus recovery under specific dynamic conditions and underscores that short-term functional mineral incorporation profiles should not be strictly generalized to long-term static solution kinetics.

Both CF-P and EF showed significant progressive increases in their absolute Ca/P ratios following pH cycling, whereas the CF + P group showed no statistically significant shift from its post-demineralization state. This baseline-adjusted divergence, supported by the significant material-by-time interaction, matches the findings of prior literature documenting consistent Ca/P increases for both alkasites and glass hybrids adjacent to demineralized substrates [47], and aligns with established use of Ca/P ratio as a mineral stoichiometry indicator [48]. As noted by Zhou et al., shifts in Ca/P ratio toward values approximating natural dentin mineral may reflect development toward a more stable calcium phosphate phase [49]. The lack of a significant Ca/P ratio change in CF + P is consistent with findings demonstrating that adhesive coatings substantially reduce ion release from bioactive materials and inhibit the formation of apatite-like surface precipitates [11]. This indicates that the presence of an adhesive interface interferes with the local ionic saturation required for mineral phase development [30, 31].

The consistent reduction in elemental mineral recovery associated with primer application across all evaluated parameters suggests the involvement of multiple inhibitory mechanisms. From a physicochemical perspective, this effect may primarily be related to the barrier properties of the polymerized adhesive interface. Following polymerization, resin-based primers form a cross-linked hybrid layer that reduces dentin permeability; this dense organic network restricts the diffusion of calcium, phosphate, and fluoride ions from the restorative material into the adjacent demineralized substrate [31, 35].

In addition, acidic functional monomers within the primer, such as 10-MDP, are capable of interacting with calcium ions at the interface [50]. This potential calcium chelation may reduce localized ionic availability and interfere with the supersaturation conditions favorable for mineral deposition [11, 50]. As established by Tay & Pashley, achieving localized ionic supersaturation is a critical thermodynamic requirement for mineral phase nucleation within demineralized dentin [51]. While these proposed diffusion- and chelation-related mechanisms are consistent with established biomaterials principles [22, 31], they should be interpreted as mechanistic hypotheses rather than direct experimental findings, as interfacial ion transport kinetics and permeability were not directly evaluated in the present study.

Supporting evidence for primer-mediated inhibition is well-documented in the literature. Previous reports identified negligible calcium gain at the restorative interface when an alkasite was paired with a bonding agent [52], corroborating the reduced mineral incorporation observed in CF + P. Resin-rich hybrid layers can significantly reduce dentin permeability [31], and the degree of this inhibition appears to be formulation-dependent and time-sensitive. Prior investigations noted that while Cention Forte with primer initially exhibited restricted ion diffusion, fluoride release surpassed that of the unprimed material by day 28 under static conditions [53], while its fluoride-recharging capacity remained intact despite reduced cumulative release [54].

These findings suggest that standard resin primers act as a kinetic barrier that delays ion mobilization without necessarily eliminating long-term bioactivity under static conditions. However, in the dynamic pH-cycling model used here, which more closely simulates intraoral acid challenges, this kinetic delay appears functionally significant—manifesting as a measurable reduction in dentin surface mineral recovery within the 14-day evaluation window. The degree of primer-mediated inhibition appears to be formulation-dependent, as different self-adhesive bioactive systems exhibit varying bond durability and ion-release characteristics [55]. It has been further demonstrated that biomimetic primers containing polyacrylic acid and sodium tripolyphosphate can actively support, rather than impede, mineral recovery [37], highlighting that the composition of the adhesive interface is a modifiable variable with direct implications for bioactive material performance.

Several limitations of the present study warrant consideration. First, the artificial in vitro demineralization model, while producing standardized and reproducible lesions, does not replicate the structural heterogeneity, microbial ecology, or biofilm dynamics of natural carious lesions. As noted by Moron et al., different demineralization protocols produce lesions with varying hardness and mineral content profiles, which may influence subsequent remineralization outcomes [56]. Second, the 14-day pH-cycling period represents an intermediate stage of mineral recovery; performance rankings may shift over longer evaluation periods as ion-release kinetics and mineral maturation evolve [43, 44, 53].

Additionally, surface microhardness and EDX elemental analysis are validated but indirect proxies for mineral recovery. The present data do not confirm crystalline hydroxyapatite formation, intrafibrillar mineral deposition, or mineral deposition depth, which would require structural characterization by X-ray diffraction, Raman spectroscopy, or transmission electron microscopy. Fourth, the proposed mechanisms for primer-mediated inhibition remain inferential, as no direct measurements of ion diffusion kinetics, interfacial permeability, or ion release profiles were performed.

Future studies should incorporate microbiological caries models, extended evaluation periods, structural mineral characterization, and depth-resolved analysis to provide a more complete assessment of bioactive material performance and to validate the mechanistic hypotheses proposed here. Investigation of modified primer formulations designed to maintain adhesion without compromising ion-release dynamics would also be of direct clinical relevance.

## Conclusion

Within the limitations of this in vitro study, all restorative protocols demonstrated significant surface microhardness recovery following pH cycling. However, application of the proprietary primer significantly reduced calcium and phosphorus recovery and attenuated changes in the Ca/P ratio. Ultimately, Cention Forte applied without primer and Equia Forte HT demonstrated statistically comparable surface mineral-related recovery behavior.

## Data Availability

The datasets generated and analyzed during the current study are available from the corresponding author upon reasonable request.

## Abbreviations

CEJ: Cementoenamel junction
CF+P: Cention Forte with primer
CF−P: Cention Forte without primer
DCP: Dicalcium phosphate
EDTA: Ethylenediaminetetraacetic acid
EDX: Energy-dispersive X-ray spectroscopy
EF: Equia Forte HT
ESEM: Environmental scanning electron microscopy
GIC: Glass ionomer cement
HEMA: 2-Hydroxyethyl methacrylate
HVGIC: High-viscosity glass ionomer cement
MDP: 10-Methacryloyloxydecyl dihydrogen phosphate
SEM: Scanning electron microscopy
UDMA: Urethane dimethacrylate
VHN: Vickers hardness number
XRD: X-ray diffraction
